# Complete genome sequence of *Nguyenibacter* sp. L1, a phosphate solubilizing bacterium isolated from *Lespedeza bicolor* rhizosphere

**DOI:** 10.3389/fmicb.2023.1257442

**Published:** 2023-12-11

**Authors:** Xiao Li Li, Xin Yang Lv, Jun Bin Ji, Wei Duo Wang, Ji Wang, Cong Wang, Hai Bin He, Ai Ling Ben, Ting Li Liu

**Affiliations:** ^1^School of Food Science, Nanjing Xiaozhuang University, Nanjing, China; ^2^College of Horticulture and Plant Protection, Henan University of Science and Technology, Luoyang, China; ^3^College of Forestry, Nanjing Forestry University, Nanjing, China

**Keywords:** *Nguyenibacter* sp. L1, whole genome, gene functions, nitrogen fixation, phosphate dissolution

## Abstract

Phosphorus (P) deficiency is a predominant constraint on plant growth in acidified soils, largely due to the sequestration of P by toxic aluminum (Al) compounds. Indigenous phosphorus-solubilizing bacteria (PSBs) capable of mobilizing Al-P in these soils hold significant promise. A novel Al-P-solubilizing strain, Al-P *Nguyenibacter* sp. L1, was isolated from the rhizosphere soil of healthy *Lespedeza bicolor* plants indigenous to acidic terrains. However, our understanding of the genomic landscape of bacterial species within the genus *Nguyenibacter* remains in its infancy. To further explore its biotechnological potentialities, we sequenced the complete genome of this strain, employing an amalgamation of Oxford Nanopore ONT and Illumina sequencing platforms. The resultant genomic sequence of *Nguyenibacter* sp. L1 manifests as a singular, circular chromosome encompassing 4,294,433 nucleotides and displaying a GC content of 66.73%. The genome was found to host 3,820 protein-coding sequences, 12 rRNAs, and 55 tRNAs. Intriguingly, annotations derived from the eggNOG and KEGG databases indicate the presence of genes affiliated with phosphorus solubilization and nitrogen fixation, including *isc*U, *gln*A, and *glt*B/D associated with nitrogen fixation, and *pqq*BC associated with inorganic phosphate dissolution. Several bioactive secondary metabolite genes in the genome, including *pqq*CDE, phytoene synthase and squalene synthase predicted by antiSMASH. Moreover, we uncovered a complete metabolic pathway for ammonia, suggesting an ammonia-affinity property inherent to *Nguyenibacter* sp. L1. This study verifies the nitrogen-fixing and phosphate-dissolving abilities of *Nguyenibacter* sp. L1 at the molecular level through genetic screening and analysis. The insights gleaned from this study offer strategic guidance for future strain enhancement and establish a strong foundation for the potential incorporation of this bacterium into agricultural practices.

## 1 Introduction

Phosphorus (P) serves as an essential macronutrient underpinning plant growth and development (Tania et al., 2010), playing a key role in numerous physiological and biochemical processes. As per the United Nations’ latest forecasts, the global population will swell to an estimated 9.7 billion by 2050 (Goswami and Deka, 2020). To satiate the escalating demand for food and shelter, the agricultural industry often resorts to the excessive use of chemical fertilizers, notably phosphate fertilizers. However, this practice often results in the inefficient utilization or immobilization of these fertilizers in the soil. Acid soils blanket approximately 30% of ice-free land and encroach upon 70% of potential arable soils worldwide (von Uexküll and Mutert, 1995). In China, acid soils stretch across 2.18 million km^2^, comprising 22.7% of the country’s total area (Huang and Zhao, 2014). Within these acid soils, most phosphates exist as insoluble iron and aluminum phosphates, rendering the majority of phosphorus from added fertilizer inaccessible to plants (Hemwall, 1957; Tian et al., 2021). Moreover, the protracted application of phosphorus fertilizers risks causing soil salinization and pollution, including groundwater contamination due to leaching and transpiration. These environmental quandaries threaten to diminish food production and potentially jeopardize human health (Wang et al., 2023). Unlike nitrogen (N), which has volatile atmospheric forms such as N_2_ and N_2_O, phosphorus lacks a soluble atmospheric supply (Yang and Post, 2017). In natural ecosystems, phosphorus primarily derives from rock weathering, cementing its status as an unrenewable resource and the world’s second-most limiting nutrient for plant growth (Walker and Syers, 1976). Therefore, unshackling soil-bound phosphorus and converting it into forms that are readily available to plants and microbes stand as paramount goals in the development and evolution of agriculture and forestry practices.

Soil microorganisms occupy a pivotal role in the facilitation of plant nutrient uptake and are the crux of an array of biological processes (Timofeeva et al., 2022). Abundant research illuminates the multifaceted capabilities of phosphate-solubilizing bacteria within the soil and rhizosphere, showcasing their potential to enhance host plant growth through mechanisms such as organic phosphate mineralization and inorganic phosphate dissolution (Li et al., 2016). These bacteria, commonly referred to as phosphate-solubilizing microorganisms (PSM), have been garnering significant attention due to their eco-friendly nature, cost-effectiveness, and high efficiency (Khan et al., 2007; Owen et al., 2015). PSM demonstrate their plant growth-promoting abilities by amplifying biological nitrogen fixation efficiency, stimulating phytohormone synthesis, and bolstering the bioavailability of specific micronutrients like zinc and iron (Wani et al., 2007). Concurrently, experiments with PSM inoculation have unequivocally evidenced increased plant yield and phosphorus uptake in both controlled pot experiments and field conditions (Wang et al., 2015). Given its economic and environmental benefits, the phosphate solubilization capacity of soil microorganisms is deemed a particularly promising research frontier. Therefore, the strategy of inoculating soil or crops with phosphate-solubilizing microorganisms is perceived as an advantageous approach to augmenting plant phosphorus uptake (Alori et al., 2012).

The advent and advancement of genetic tools and technology, particularly whole genome sequencing, have opened up promising avenues for investigating the physiological and ecological functions of rhizobacteria (Jana and Yaish, 2021). For instance, Soni et al. (2021) probed the draft genome of *Paenibacillus polymyxa* HK4, successfully identifying genes associated with phosphorus solubility and confirming its abilities in biological control and plant growth promotion. Similarly, Xu et al. (2022) shed light on the genetic attributes of *Rahnella aceris* ZF458 pertaining to plant growth, utilizing genome annotation and comparative genomics. This work laid the groundwork for investigating genes associated with phosphorus solubility. Leveraging molecular biology techniques allows the extraction of functional genes involved in siderophore synthesis, nitrogen fixation, and phosphorus dissolution. These genes play pivotal roles in elucidating key growth-promoting attributes (Cleton-Jansen et al., 1991; Straub et al., 2013; Kang et al., 2016; Silva et al., 2020). Therefore, the process of mining these functional genes is not only valuable but also instrumental in bolstering research on the growth-promoting traits of bacterial strains. It offers insights into the metabolic pathways of these strains and can potentially enhance the applicability and efficiency of PSM in plant growth promotion.

Within our laboratory, strain L1 has been identified as exhibiting high AlPO4 solubilizing capability and was initially classified as *Nguyenibacter* based on its 16S rRNA gene sequence (Li Q. et al., 2021). However, given the paucity of genomic information available for this species, a more comprehensive molecular examination of strain L1 is warranted.

Prior research indicated that the combined inoculation of two strains, L1 and S1, significantly bolstered soybean growth under greenhouse conditions (Li et al., 2023). Furthermore, previous studies proposed that *Nguyenibacter* possesses N-fixing capabilities (Thi Lan Vu et al., 2013). Nonetheless, the metabolic pathways and mechanisms underlying these functions remain enigmatic. We hypothesize that the biological control and plant growth-promoting attributes of strain L1 are intrinsically linked to key growth-promoting genes. Consequently, we undertook the sequencing of the L1 genome with the aim of unearthing and verifying its plethora of gene functions associated with growth-promoting properties and capabilities. The data generated from this investigation enriches our understanding of plant growth-promoting mechanisms, casting fresh light on the operative processes within this realm.

## 2 Materials and methods

### 2.1 Bacterial strain and DNA preparation

*Nguyenibacter* sp. L1 was isolated from the rhizosphere soil surrounding wild *Lespedeza bicolor* plants, located in an acidic soil environment at the Yingtan Red Soil Ecological Experimental Station (28°40′ N, 117°03′ E), China. This strain was isolated on October 11, 2018, and subsequently deposited in the China Center for Type Culture Collection (CCTCC) under the accession number M2021392. Standard cultivation of this strain involved incubation in Luria-Bertani (LB) liquid media, held at a constant temperature of 30°C, and subjected to periodic shaking over a duration of 48 h (h). The extraction of the genomic DNA was facilitated using the Tiangen Bacterial Genomic DNA Extraction Kit (Tiangen Biotech, Beijing, China).

### 2.2 DNA library preparation, genome sequencing and assembly

The whole genome of *Nguyenibacter* sp. L1 was sequenced using the Illumina NovaSeq sequencing platform (Illumina, San Diego, CA). A Whole Genome Shotgun (WGS) strategy was adopted to construct various insert libraries, which were then sequenced using both next-generation sequencing (NGS) on the Illumina HiSeq sequencing platform and third-generation single-molecule sequencing technology on the PacBio Sequel sequencing platform. Subsequent assembly of the third-generation sequencing data was carried out using HGAP3 (Chen et al., 2016) and CANU1.8 (Koren et al., 2017). Pilon1.23 was then employed to apply corrections from the high-quality next-generation data to the third-generation contig results (Walker et al., 2014), thereby yielding the complete sequence. The complete procedures for whole-genome sequencing and assembly were executed by Personal Gene Technology Co., Ltd. (Nanjing, China). The sequencing data pertinent to this publication has been deposited in the NCBI’s short read archive under the accession number PRJNA975354.

### 2.3 Open reading frames, carbohydrate-active enzymes analysis, gene functional annotation and secondary metabolite biosynthesis gene clusters of *Nguyenibacter* sp. L1

For open reading frames (ORFs) prediction, GeneMarkS software was utilized (Besemer et al., 2001). The prediction of carbohydrate-active enzymes was conducted using CAZy software. The predicted gene sequences were then compared with various functional databases, including evolutionary genealogy of genes: Non-supervised Orthologous Groups (eggNOG), KEGG, Swiss-Prot, TrEMBL, nonredundant (Nr), and other functional databases, using BLAST. This enabled the acquisition of gene functional annotations (Tatusov et al., 2003). The comparison results from the Nr database (Aziz et al., 2008) facilitated functional annotation according to the Gene Ontology (GO) database using the application software Blast2GO. For functional annotation based on the Pfam database, the HMMER software was employed (El-Gebali et al., 2019). In addition, gene functions were annotated and analyzed through the utilization of eggNOG and Kyoto Encyclopedia of Genes and Genomes (KEGG) metabolic pathway enrichment analyses. Bacterial secondary metabolite biosynthesis gene clusters were identified and annotated with antiSMASH 6.0.^1^

## 3 Results

### 3.1 General genome characteristics of *Nguyenibacter* sp. L1

**Figure 1:**
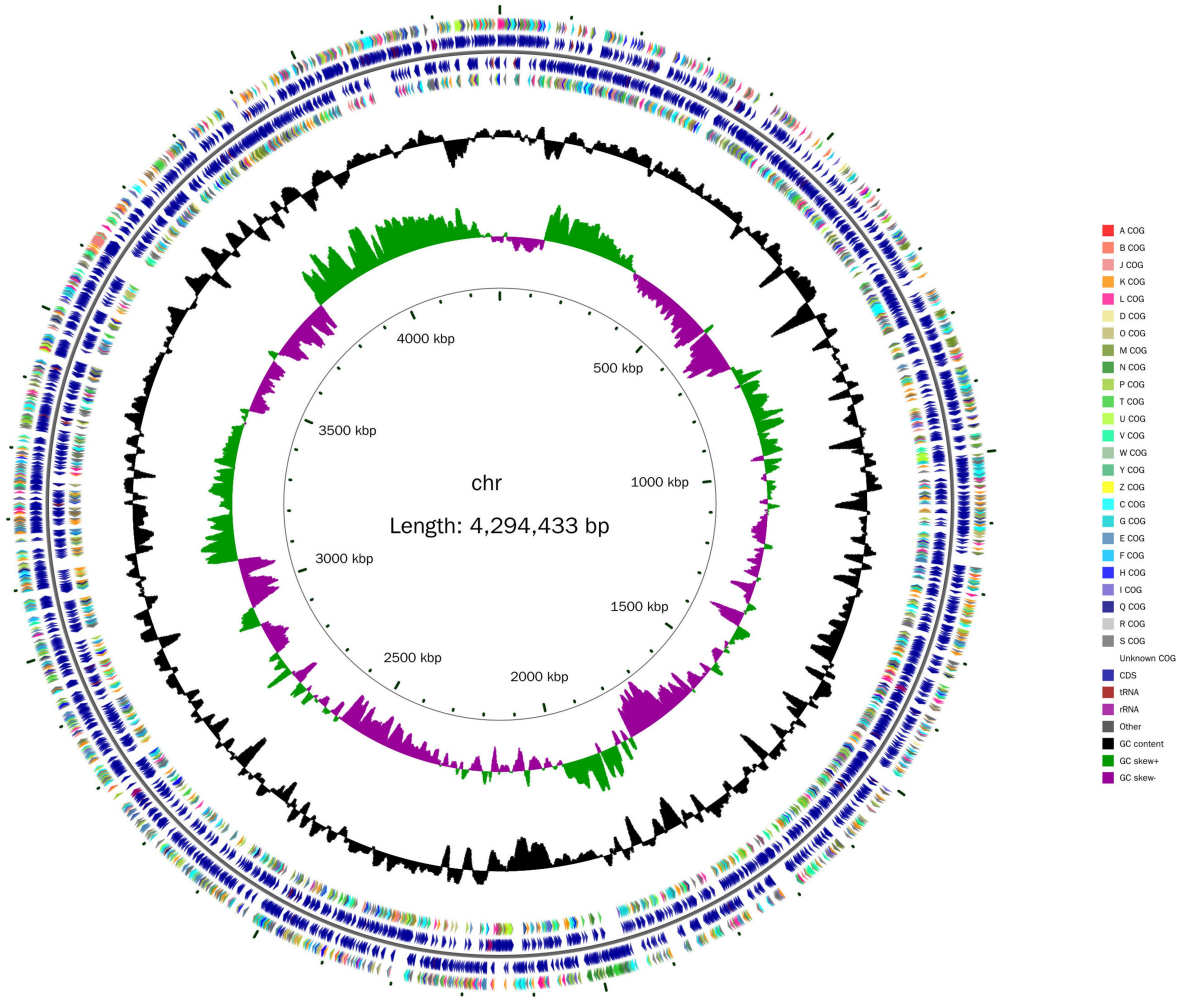
Schematic representation of the genomic organization of *Nguyenibacter* sp. L1. The circle, from centre to the outside, represents the scale mark, the second circle represents GC skew, the third circle corresponds to GC content, the fourth and seventh circles represent every COG to which each coding sequence (CDS) belongs, and the fifth and sixth circles represent the locations of CDS, tRNA and rRNA in the genome.

Whole-genome sequencing revealed that the *Nguyenibacter* sp. L1 genome comprises a single chromosome of 5,328,700 bp with a GC content of 66.73% (Table 1). This chromosome harbors 3,820 ORFs. Furthermore, the prediction of noncoding RNAs (ncRNAs) revealed 12 ribosomal RNAs (rRNAs), 55 transfer RNAs (tRNAs), and 12 other types of ncRNAs. The genome circle map of *Nguyenibacter* sp. L1 is depicted in Figure 1. A total of 3,331 protein-coding genes were predicted, with a significant proportion annotated to various functional categories: 87.20% to eggNOG functional categories, 75.63% to GO functional categories, and 49.76% to KEGG pathways. Detailed information is presented in Table 1.

**Table 1:** Genomic characteristics of *Nguyenibacter* sp. L1.

Genomic Contents	Chromosome
Sequence length (bp)	4,294,433
GC content (%)	66.73
Open reading frames	3,820
Number of tRNAs	55
Number of 5rRNAs	4
Number of 16rRNAs	4
Number of 23rRNAs	4
Number of ncRNAs	12
Genes assigned to eggNOG	87.20%
Genes assigned to KEGG	75.63%
Genes assigned to GO	49.76%

### 3.2 Gene function annotation of *Nguyenibacter* sp. L1 and carbohydrate-active enzymes database

**Figure 2:**
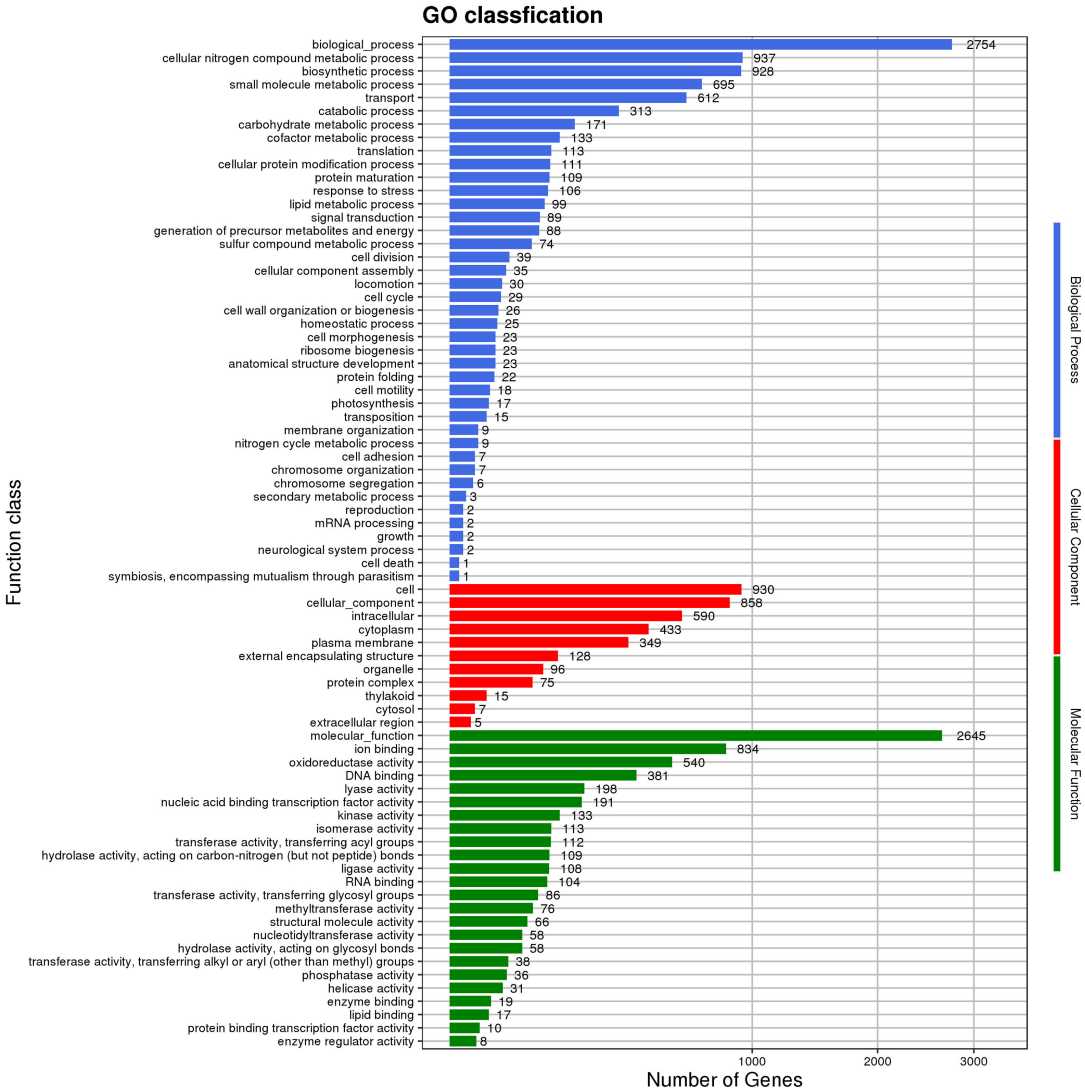
Depiction of Gene ontology (GO) functional annotation: classification distribution.

**Figure 3:**
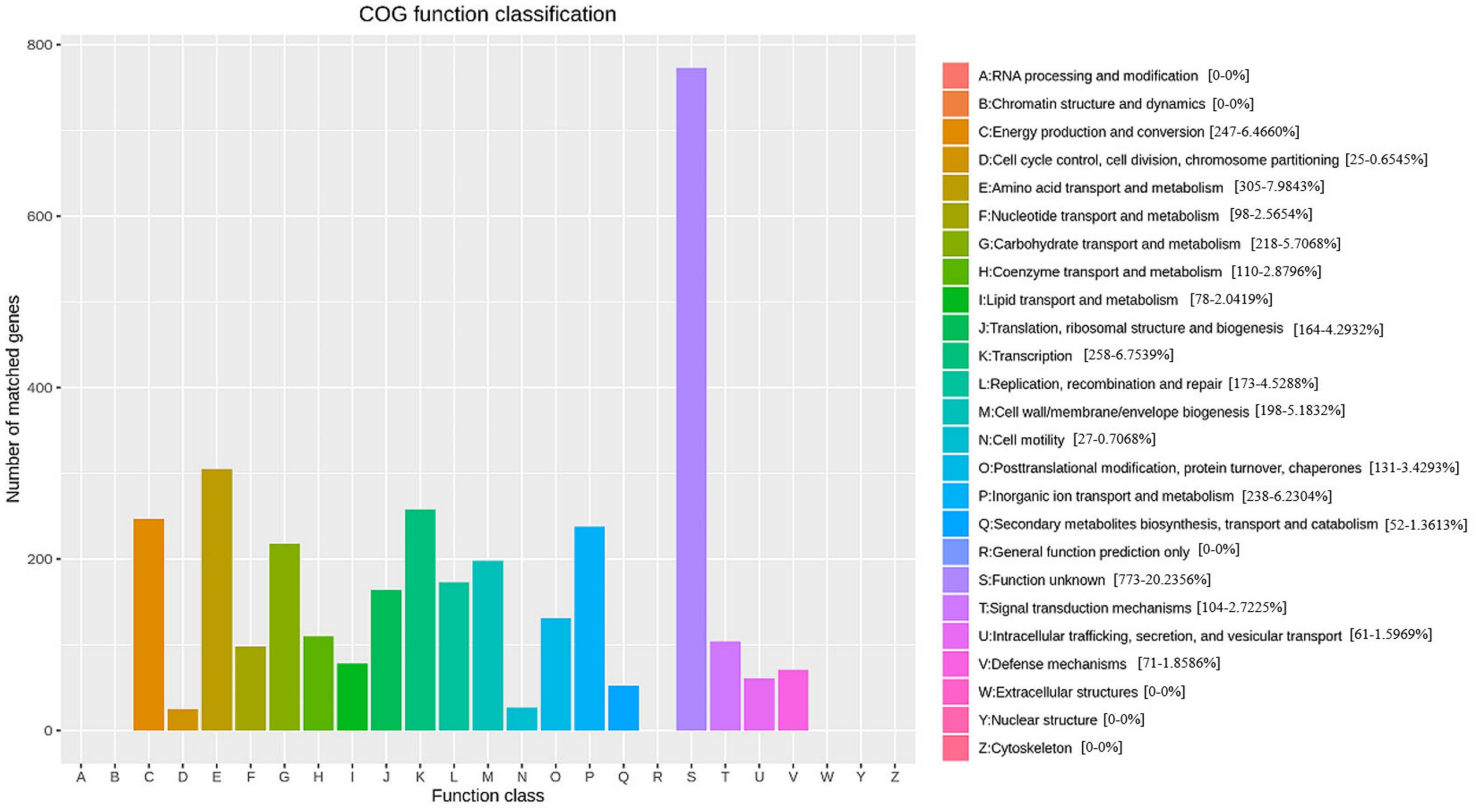
Evoluationary Genealogy of Genes: Non-supervised Orthologous Groups (eggNOG /COG) functional annotation overview.

GO annotations disclosed that the gene functions of *Nguyenibacter* sp. L1 predominantly align with biological process (BP; Figure 2; Supplementary Table S1). This includes 937 genes implicated in cellular nitrogen metabolic processes (Supplementary Table S2), 928 genes involved in biosynthetic processes (Supplementary Table S3), and 695 genes participating in small molecule metabolic processes (Supplementary Table S4). Additionally, 612 genes associated with transport responses were identified (Supplementary Table S5). Annotations allocating distinct sequences to various eggNOG categories revealed a maximum enrichment of 305 genes in the E category (Amino acid transport and metabolism). This category includes genes predicted to encode phosphoribosyl-ATP pyrophosphatase and oxidoreductase, among others. Out of the 3,331 genes encoding proteins, 19 different types were discerned. Among these, 305 genes coded for proteins related to amino acid transport and metabolism (E), 218 genes were tasked with carbohydrate transport and metabolism (G), and 258 genes encoded transcription-related proteins (K; Figure 3). This suggests that the functional proteins encoded by the cell genome play an important role in sustaining cellular life and genetic metabolism (Supplementary Table S6). Moreover, 52 genes connected to the biosynthesis, transport, and catabolism of secondary metabolites were identified. These include various organic acids implicated in the dissolution of inorganic phosphorus. There are also genes with unknown functions (S) that await further exploration.

**Figure 4:**
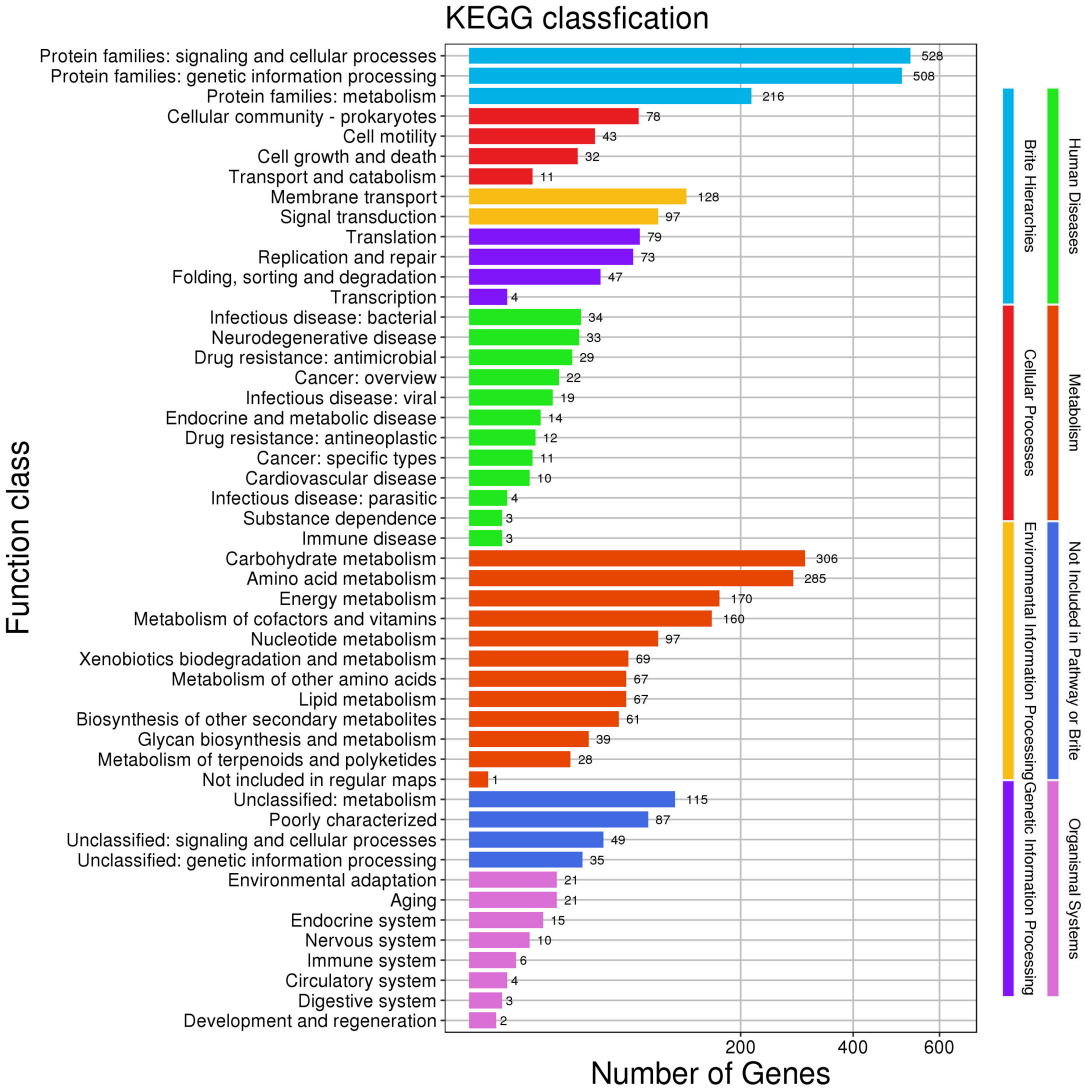
Annotation derived from Kyoto encyclopedia of genes and genomes (KEGG): cluster distribution.

KEGG annotations enabled the prediction of 49 functional classes in the genome of *Nguyenibacter* sp. L1. These can be organized into eight categories: (1) Brite Hierarchies, including signaling and cellular processes (528); (2) Cellular Processes, such as cell motility (43) and transport and catabolism (11); (3) Environmental Information Processing, which includes membrane transport (128) and signal transduction (97); (4) Genetic Information Processing; (5) Immunity; (6) Metabolism; (7) Not Included in Pathway or Brite; and (8) Organismal Systems. Of these, the metabolism-related systems constituted the largest segment of the metabolic pathways in *Nguyenibacter* sp. L1 (Figure 4). The above results imply the robust genetic potential of the *Nguyenibacter* sp. L1 strain for synthesizing secondary metabolites, corroborating the proceeding protein annotation findings.

**Figure 5:**
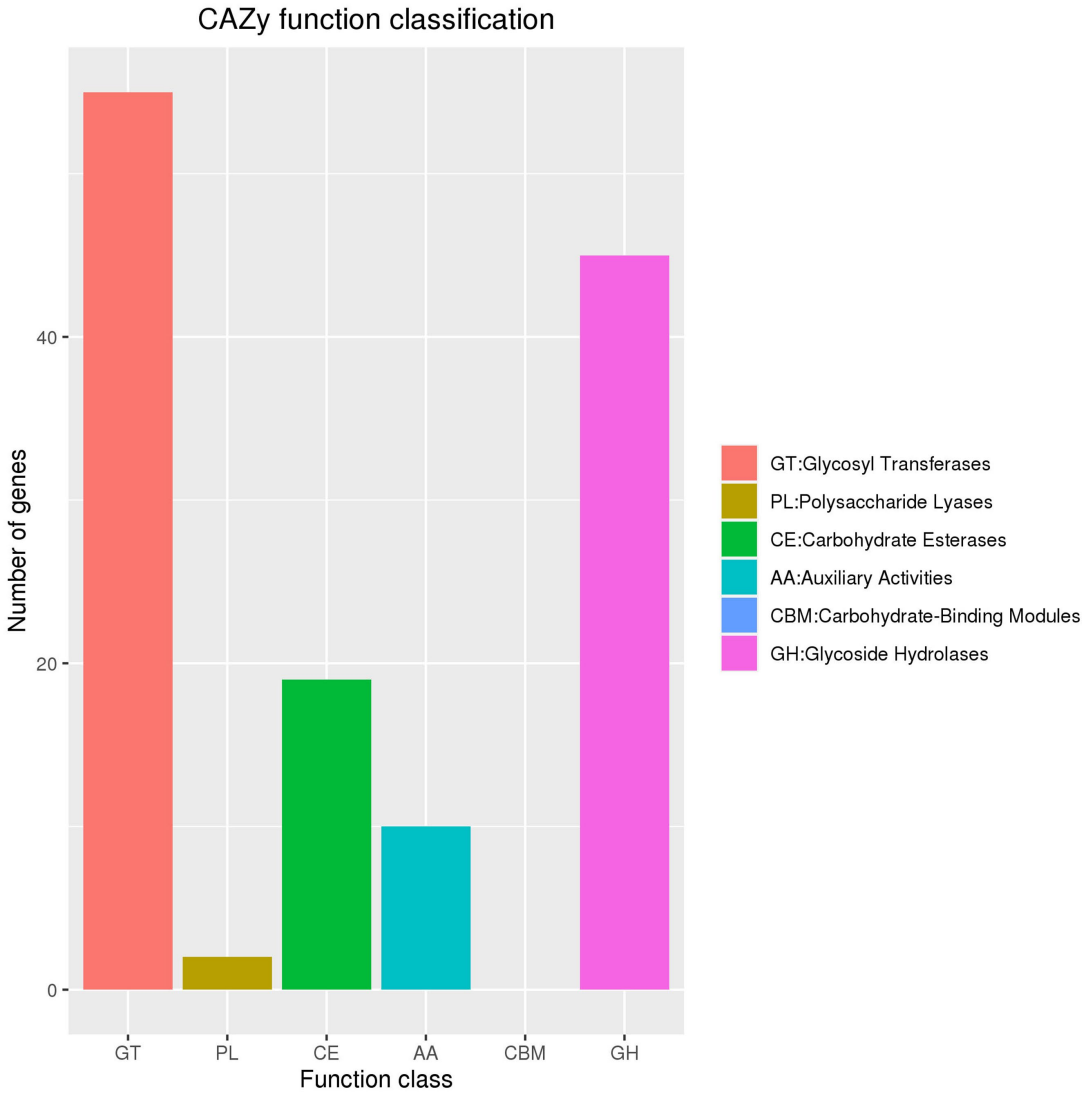
Carbohydrate-active enzymes in *Nguyenibacter* sp. L1.

Exploration of the carbohydrate-active enzyme database revealed 55 ORFs annotated as glycosyl transferases genes, two as polysaccharide lyase genes, 19 as carbohydrate esterase genes, 10 as auxiliary activities genes, and 45 as glycoside hydrolase genes. However, no genes were identified as carbohydrate-binding modules (Figure 5).

### 3.3 Nitrogen metabolic pathway

**Figure 6:**
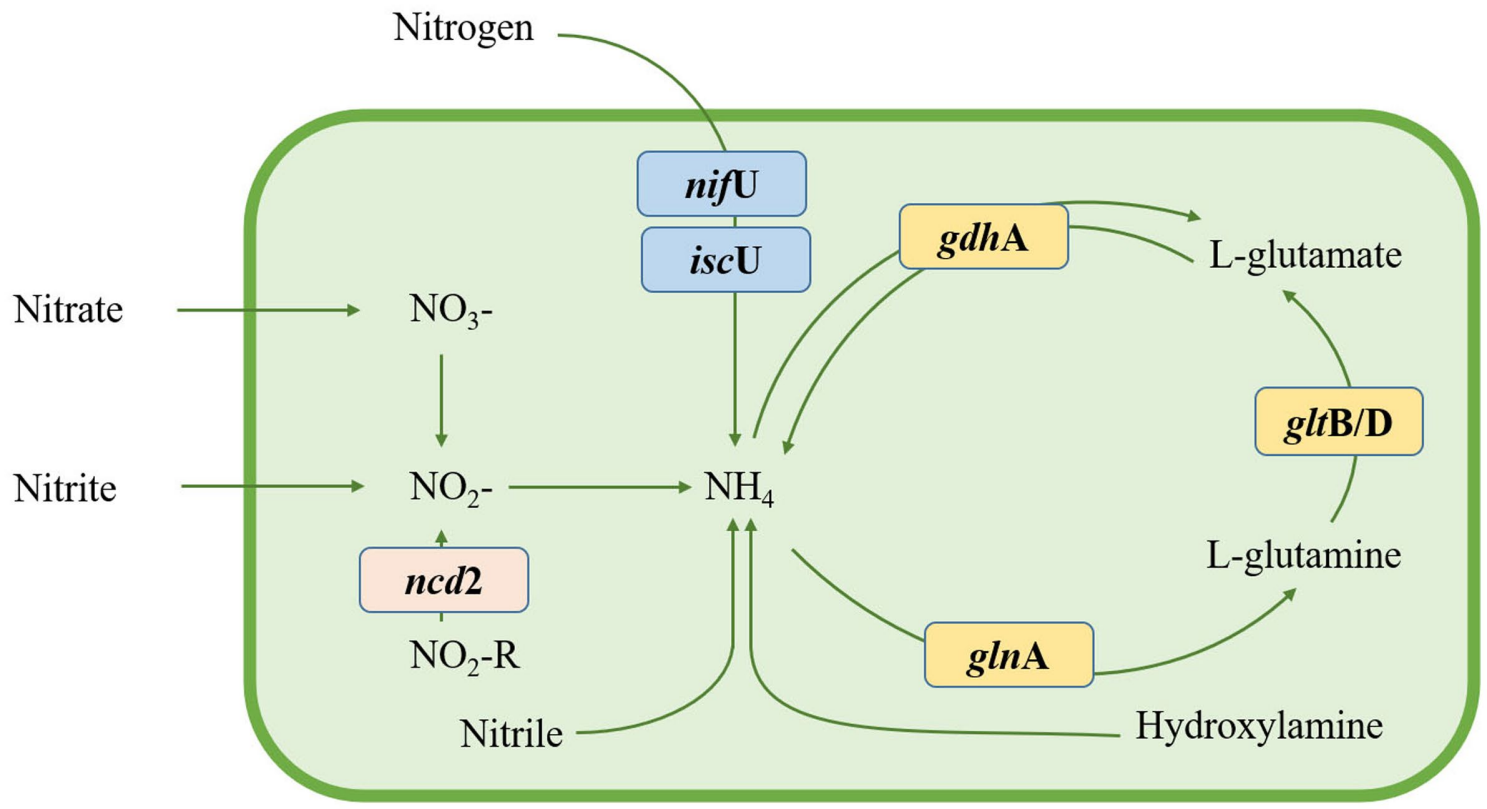
Nitrogen metabolic pathway in *Nguyenibacter* sp. L1 based on the Kyoto encyclopedia of genes and genomes (KEGG) annotations.

Nitrogen, a vital nutrient for plant growth, primarily exists as atmospheric nitrogen (N_2_), which plants struggle to directly utilize. Plant growth-promoting rhizobacteria (PGPR) can possess the ability to convert atmospheric N_2_ to NH^4+^ through the nitrogenase complex (Hofman et al., 2014). In the genome of *Nguyenibacter* sp. L1, we identified two genes related to nitrogen fixation (*isc*U and *nif*U; Figure 6). *Nif*U is generally dedicated to the biogenesis of the nitrogenase iron-sulfur (Fe-S) cluster in diazotrophs (Li X. L. et al., 2021). Fe-S clusters are contained in a diverse group of proteins called Fe-S proteins, which participate in a wide variety of cellular processes (Braymer and Lill, 2017; Braymer et al., 2021), such as nitrogen fixation, respiration, DNA repair, and gene regulation. The dimeric protein *isc*U contributes to the assembly of iron–sulfur clusters in the nitrogen fixation pathway, while gltB/D encodes glutamate synthase genes, among others. These genes together complete the metabolic pathway for ammonia, suggesting an adaptive favorability towards ammonia in *Nguyenibacter* sp. L1.

### 3.4 Gene mining for insoluble phosphate activated by *Nguyenibacter* sp. L1

It is well recognized that a substantial proportion of inorganic phosphates become immobilized after their application as fertilizers, which consequently renders the phosphate inaccessible to plants (Xie et al., 2016). To combat this issue, certain bacterial species secrete substantial amounts of acid phosphatases and organic acids, predominately gluconic acid, to solubilize these insoluble or sparingly soluble mineral phosphates (Rodríguez et al., 2007). When inoculated into growth medium, *Nguyenibacter* sp. L1 secretes significantly higher quantities of gluconic acid relative to other organic acids, thereby aiding the solubilization of Al-P (Li et al., 2021). Gluconic acid, a type of organic acid, assists in transforming the immobilized mineral phosphates (which contain phosphorus) into a form that is readily available for biological uptake. The enzymatic synthesis of gluconic acid is catalyzed by glucose-1-dehydrogenase (*gcd*) with the assistance of its cofactor, pyrrolo-quinolone quinine (*pqq*). Additionally, gluconate dehydrogenase serves a dual role in the production of gluconic acid and its conversion into 2-ketogluconate (Ramachandran et al., 2006; Eastman et al., 2014; Yu et al., 2022). On inspecting the genomic annotation of *Nguyenibacter* sp. L1, we identified 16 genes related to the synthesis of organic acids, phosphatases, and transport systems of inorganic phosphorus (Table 2). We observed three genes (*phn*P, *glp*Q, and *ugp*Q) annotated in the process of organic phosphoester hydrolysis. Among these, *ugp*Q and *glp*Q encode for periplasmic glycerophosphoryl diester phosphodiesterase. The genes primarily responsible for inorganic acid synthesis include the gcd gene, which encodes GDH, and the pqqBC gene, involved in the synthesis of PQQ. Our previous organic acid component analysis indicated that gluconic acid was the principal organic acid responsible for phosphate solubilization in L1 (Li Q. et al., 2021). Before they can be metabolized, phosphorus compounds must be translocated across the plasma membrane. This is facilitated by a high-affinity phosphate transport system, *pst*SBAC (phosphate transporter; Zeng et al., 2022), also detected in strain L1, along with the phosphonate transporter (Table 2).

**Table 2:** Annotation of phosphorus soluble-related genes in *Nguyenibacter* sp. L1.

Cyclic process		ID	Key genes	Function annotation
Phosphorus nutrient cycling	Organic phosphate mineralization	K06167	*phn*P	Phosphate phosphodiesterase
		K01126	*glp*Q/*ugp*Q	Glycerophosphoryl diester phosphodiesterase
	Inorganic phosphate dissolution	K01507	*ppa*	Inorganic pyrophosphatase
		K01524	*ppx*	Exopolyphosphatase/guanosine-5′-triphosphate,3′-diphosphate pyrophosphatase
		K00117	*gcd*	Quinoprotein glucose dehydrogenase
		K06137	*pqq*C	Pyrroloquinoline-quinone synthase
		K06136	*pqqB*	Pyrroloquinoline quinone biosynthesis protein
		K00937	*ppk*	Polyphosphate kinase
		K22468	*ppk2*	Polyphosphate kinase
	Regulatory	K07657	*pho*B	Phosphate regulon response regulator
		K07636	*pho*R	Phosphate regulon sensor histidine kinase
	Transporters	K02040	*pst*S	Phosphate transport system substrate-binding protein
		K02036	*pst*B	Phosphate transport system ATP-binding protein
		K02038	*pst*A	Phosphate transport system permease protein
		K02037	*pst*C	Phosphate transport system permease protein
		K03306	*pit*	Inorganic phosphate transporter

### 3.5 Secondary biosynthesis metabolite clusters of *Nguyenibacter* sp. L1

Bioinformatic analysis using the antiSMASH website provided secondary metabolite prediction. The results show that cluster 2 belonged to the biosynthesis gene cluster (BGC), including *pqq*CED (Table 3). Gluconic acid is synthesized by the interaction between co-factor pyrroloquinoline quinine (PQQ) and glucose dehydrogenase within themselves for converting the insoluble phosphate into a soluble form (Bhanja et al., 2021). The results have revealed that this genus has potential as producing secondary metabolite about the solubilization of inorganic phosphates.

**Table 3:** The secondary biosynthesis metabolite of *Nguyenibacter* sp. L1 predicted by antiSMASH.

Clusters	Type of secondary metabolite	gene	Start site	Termination site	Protein
Cluster-1	RiPP-like	gene1801	2,033,829	2,034,654	Encapsulin nanocompartment shell protein
Cluster-2	Redox-cofactor	gene2129	2,404,646	2,405,384	Pyrroloquinoline-quinone synthase *pqq*c
		gene2130	2,405,380	2,405,674	Pyrroloquinoline quinone biosynthesis peptide chaperone *pqq*d
		gene2131	2,405,670	2,406,765	Pyrroloquinoline quinone biosynthesis protein *pqq*e
Cluster-3	Terpene	gene2243	2,520,672	2,522,700	Squalene-hopene cyclase
		gene2245	2,524,066	2,524,984	Phytoene synthase
		gene2246	2,525,147	2,526,110	Squalene synthase

## 4 Discussion

Plant-associated endophytic microbes have intricately evolved their biosynthetic pathways to facilitate symbiotic interactions with their hosts. The diverse metabolites derived from these endophytes contribute to plant growth promotion, thereby propelling significant interest towards their genomic studies in recent years (Patel et al., 2016; Kandel et al., 2022). *Nguyenibacter*, for instance, is documented as an N-fixing bacterium (Thi Lan Vu et al., 2013). An examination of the nitrogen metabolic pathway in *Nguyenibacter* sp. L1 reveals the bacterium’s proclivity towards ammonium, bolstered by the presence of nitrogen-fixing genes (*isc*U and *nif*U), and its capacity for ammonia metabolism. Our prior research led to the isolation of *Nguyenibacter* sp. L1, which displayed notable plant growth-promoting characteristics under low-phosphorus conditions (Li et al., 2023). The exploration and understanding of these inherent genes might pave the way for the development of commercial bioinoculants, potentially reducing the reliance on chemical fertilizers.

Phosphorus, an essential nutrient for plants, often emerges as a significant limiting factor for plant growth and development due to its low bioavailability (Zeng et al., 2017; Li et al., 2018). In this context, PSM play a pivotal role in the ecosystem as they catalyze the conversion of insoluble soil phosphates into forms accessible to plants, thereby mitigating the phosphorus deficit (Kour et al., 2021). *Nguyenibacter* sp. L1 was initially isolated based on its exceptional ability to solubilize AlPO4. Remarkably, when co-inoculated with *Pseudomonas* sp. S1, it exhibited considerable potential to enhance the total phosphorus content in soybean plants (Li et al., 2023). Despite these promising attributes, the intricacies of the mechanism underlying the phosphorus dissolution orchestrated by *Nguyenibacter* sp. L1 remain elusive. A comprehensive understanding of this mechanism could reveal significant insights into the sustainable management of phosphorus in agriculture.

Initial characterization of *Nguyenibacter* sp. L1 uncovered its robust ability to secrete gluconic acid, particularly in the presence of glucose as a carbon source (Li X. L. et al., 2021). In the present study, genome analysis of *Nguyenibacter* sp. L1 offered intriguing hints to the presence of distinct genes instrumental for phosphate solubilization. PSM enhances plant growth by transforming mineral phosphate into a form readily absorbed by plants. This is achieved via an array of solubilization mechanisms, including the production of extracellular enzymes and the release of organic acids, protons, and hydroxyl ions. Phosphate-solubilizing bacteria such as *Bacillus*, *Pseudomonas*, and *Pantoea* are renowned for their gluconic acid secretion (Rodríguez et al., 2007). It is noteworthy that *Enterobacter asburiae* mutants lacking glucose dehydrogenase (GDH) activity failed to solubilize phosphate (Gyaneshwar et al., 1999). GDH plays a pivotal role in the periplasmic oxidation of glucose to gluconic acid, a major metabolic pathway involved in inorganic phosphate solubilization in Gram-negative bacteria (Goldstein, 1994; de Werra et al., 2009). In the *Nguyenibacter* sp. L1 genome, we identified the presence of *gcd* and *pqq* genes, which are critical for the synthesis of gluconic acid and the solubilization of inorganic mineral phosphates. Meanwhile, secondary metabolite genes *pqq*CDE in the genome were predicted by antiSMASH. The *gcd* gene, encoding an enzyme that mediates inorganic P solubilization, was predominant across soil samples and was a major determinant of bioavailable soil P (Liang et al., 2020). These findings echo the previously reported high gluconic acid secretion capacity of this strain, thereby supporting the notion that *Nguyenibacter* sp. L1 could be a valuable biological tool in enhancing phosphate utilization in agriculture.

Microorganisms have adeptly evolved strategies to utilize alternative sources of phosphorus, such as phosphonates, particularly during phosphate scarcity. The enzymes pivotal to phosphonate catabolic pathways are conserved and encoded by orthologous genes in bacteria. *Nguyenibacter* sp. L1 harbors *phn* genes like *phn*P, integral to phosphate solubilization. Furthermore, the *ugp*Q and *glp*Q genes encode cytoplasmic glycerophosphoryl diester phosphodiesterase and periplasmic glycerophosphoryl diester phosphodiesterase, respectively, which facilitate the hydrolysis of organic phosphorus (Zeng et al., 2022). Notably, *Nguyenibacter* sp. L1 also possesses *pst*ABCS genes, representing a prominent transporter system for inorganic phosphorus uptake (Hsieh and Wanner, 2010). Comparable gene clusters have also been reported for phosphonate binding, transport, and degradation in *Rahnella victoriana* JZ-GX1 and *Pantoea agglomerans* strain P5 (Shariati et al., 2017; Kong et al., 2022).

## 5 Conclusion

This study represents the inaugural whole-genome sequencing of *Nguyenibacter* sp. L1 and genome-wide analysis of the genes associated with plant growth promotion, such as nitrogen fixation and phosphorus dissolution. The genetic screening and analysis conducted in this study offer significant insights into the functioning and potential enhancement of *Nguyenibacter*. This, in turn, might pave the way for practical applications of the stain as a biofertilizer in agriculture, thereby providing a sustainable and environmentally friendly alternative to chemical fertilizers.

## Data availability statement

The datasets presented in this study can be found in online repositories. The names of the repository/repositories and accession number(s) can be found at: https://www.ncbi.nlm.nih.gov/, PRJNA975354.

## Author contributions

XLi: Conceptualization, Data curation, Formal analysis, Investigation, Methodology, Writing – original draft. XLv: Data curation, Investigation, Writing – review & editing. JJ: Writing – review & editing. WW: Writing – review & editing. JW: Supervision, Writing – review & editing. CW: Data curation, Investigation, Visualization, Writing – review & editing. HH: Data curation, Investigation, Writing – review & editing. AB: Funding acquisition, Supervision, Writing – review & editing. TL: Conceptualization, Funding acquisition, Project administration, Resources, Supervision, Writing – review & editing.

## Footnotes

^1^https://antismash.secondarymetabolites.org
